# Position-specific automated processing of V3 *env* ultra-deep pyrosequencing data for predicting HIV-1 tropism

**DOI:** 10.1038/srep16944

**Published:** 2015-11-20

**Authors:** Nicolas Jeanne, Adrien Saliou, Romain Carcenac, Caroline Lefebvre, Martine Dubois, Michelle Cazabat, Florence Nicot, Claire Loiseau, Stéphanie Raymond, Jacques Izopet, Pierre Delobel

**Affiliations:** 1 Laboratoire de Virologie, Hôpital Purpan, Toulouse, F-31300 France; 2 INSERM, UMR1043, Toulouse, F-31300 France; 3 Université Toulouse III Paul Sabatier, Toulouse, F-31000 France; 4 Service des Maladies Infectieuses et Tropicales, Hôpital Purpan, Toulouse, F-31300 France

## Abstract

HIV-1 coreceptor usage must be accurately determined before starting CCR5 antagonist-based treatment as the presence of undetected minor CXCR4-using variants can cause subsequent virological failure. Ultra-deep pyrosequencing of HIV-1 V3 *env* allows to detect low levels of CXCR4-using variants that current genotypic approaches miss. However, the computation of the mass of sequence data and the need to identify true minor variants while excluding artifactual sequences generated during amplification and ultra-deep pyrosequencing is rate-limiting. Arbitrary fixed cut-offs below which minor variants are discarded are currently used but the errors generated during ultra-deep pyrosequencing are sequence-dependant rather than random. We have developed an automated processing of HIV-1 V3 *env* ultra-deep pyrosequencing data that uses biological filters to discard artifactual or non-functional V3 sequences followed by statistical filters to determine position-specific sensitivity thresholds, rather than arbitrary fixed cut-offs. It allows to retain authentic sequences with point mutations at V3 positions of interest and discard artifactual ones with accurate sensitivity thresholds.

Human immunodeficiency virus type 1 enters CD4-expressing cells using one or both of the host cell coreceptors, CCR5 and CXCR4^1^,^2^,^3^. Virus strains that specifically use CCR5 or CXCR4 are termed R5 or X4 variants, while those that use both coreceptors are termed dual/mixed variants (D/M)^4^.

Maraviroc is the first CCR5 antagonist approved for treating HIV-1 infections^5^. But the HIV-1 coreceptor usage must be determined to establish that a patient is not harboring CXCR4-using viruses and is thus eligible for CCR5 antagonist treatment^6^,^7^.

Recombinant virus phenotypic entry assays are now considered to be the gold-standard for determining HIV-1 tropism^8^,^9^,^10^,^11^,^12^,^13^. These assays can detect minor CXCR4-using variants down to 0.3–0.5% of the virus population^8^,^14^. However, their routine use is hampered by technical and cost limitations. Simple alternative genotypic approaches have been developed to infer virus tropism from the V3 *env* amino acid sequence^15^,^16^,^17^,^18^,^19^. Particularly, the presence of basic residues at V3 positions 11 and/or 25 and an increased net electrostatic charge of V3 have been associated with CXCR4 usage^15^,^20^,^21^. Genotypic algorithms based on the V3 *env* sequence perform well for predicting virus tropism when they are used at a clonal level^21^. However, direct sequencing of bulk PCR products of V3 *env* at a population level cannot detect minor CXCR4-using viruses that account for less than about 20% of the quasispecies^21^,^22^,^23^. Failure to detect CXCR4-using variants initially present at low frequencies in the virus population may lead to their subsequent selection under CCR5 antagonist-based treatment^24^,^25^. Thus, there is a need for new genotypic techniques for determining tropism that are sensitive enough to detect minor CXCR4-using variants.

The sequencing of V3 *env* amplicons at high coverage with long read lengths has made massive parallel amplicon pyrosequencing using the 454 technology a promising tool for studying the virus diversity in clinical samples. It competes with ultra-sensitive phenotypic approaches for detecting low levels of CXCR4-using variants that current genotypic approaches miss, while being able to quantify the proportion of each variant in the virus quasispecies^24^,^26^,^27^,^28^,^29^,^30^,^31^,^32^,^33^,^34^,^35^,^36^.

However, processing the mass of sequence data and the need to identify true minor variants while excluding artifactual sequences is the rate-limiting step in the process. Arbitrary fixed cut-offs (1–2%) are currently used, below which minor variants are discarded, but the errors generated during ultra-deep pyrosequencing are sequence-dependant rather than random, notably in homopolymeric regions^37^.

We have developed an automated position-specific processing of V3 *env* ultra-deep pyrosequencing data for rapidly inferring HIV-1 tropism with improved detection of minor variants (PyroVir software). It uses a sequence of logic rules based on the V3 sequence to discard artifactual or non-functional sequences with frame shifts or stop codons (biological filters), followed by a position-specific matrix based on Poisson distribution (statistical filter) to discard sequences with artifactual point mutations at V3 positions of interest. A particular attention had also been paid to provide a representative description of the virus quasispecies by limiting sampling and amplification bias prior to ultra-deep pyrosequencing.

## Results

### Optimized amplification steps before ultra-deep pyrosequencing for accurate representation of HIV-1 quasispecies

We determined experimentally the number of PCR cycles for which the amplification of a given input of virus copies remains linear without distorting the proportions of minor and major variants in the quasispecies. We found that 34 cycles of RT-PCR for an input of 2,000–3,000 copies followed by 25 cycles of nested PCR were adequate to get high sensitivity without biasing the proportions in the virus population (Supplementary Fig. S1).

### Performances of ultra-deep pyrosequencing for detecting CXCR4-using variants in HIV-1 quasispecies compared to an ultrasensitive phenotypic assay

Three artificial mixtures of culture supernatants of pure X4 and R5 clones (LAI:JR-CSF; AFG4:AFG1, CHS2:CHS11) with defined proportions of X4:R5 viruses (0:100; 0.5:99.5; 1:99; 5:95; 20:80; 50:50; 75:25; and 100:0) were submitted in parallel to ultra-deep pyrosequencing and phenotyping. The TTT phenotypic assay detected 0.5% of X4 viruses in the LAI:JR-CSF mixture (1/3 replicates), 0.5% of X4 viruses in the AFG4:AFG1 mixture (1/3 replicates), and 0.5% of X4 viruses in the CHS2:CHS11 mixture (3/3 replicates). Ultra-deep pyrosequencing of the same mixtures detected 0.5% of X4 viruses in the LAI:JR-CSF mixture (2/3 replicates), 0.5% of X4 viruses in the AFG4:AFG1 mixture (2/3 replicates), and 1% of X4 viruses in the CHS2:CHS11 mixture (2/3 replicates). Our optimized process of amplification before ultra-deep pyrosequencing thus accurately described the HIV-1 quasispecies, with a 0.5–1% sensitivity for detecting CXCR4-using variants without distorting the proportions in the virus population (Table 1).

### Automated data cleaning of errors occurring during the ultra-deep pyrosequencing process

Ultra-deep pyrosequencing can generate errors that are sequence-dependant, notably in homopolymeric regions, rather than random. Our automated approach distinguishes authentic variants from artifactual ones resulting from errors arising during PCR amplification and ultra-deep pyrosequencing.

### Biological filters to discard non-functional V3 sequences

**Figure 1:**
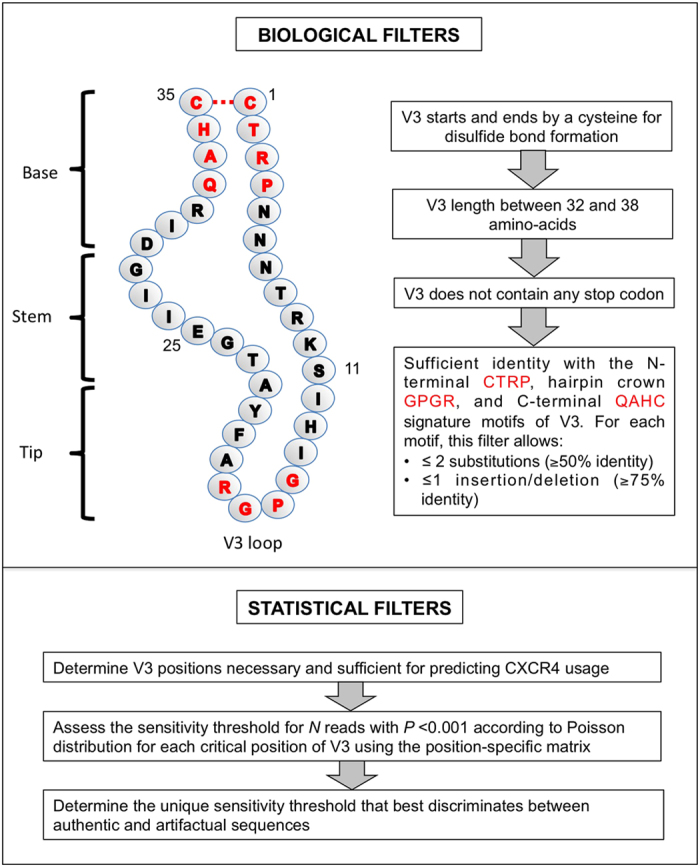
PyroVir flowchart.

The sequences from the 454 ultra-deep pyrosequencing data were first processed with GS Amplicon Variant Analyzer (AVA) software (Roche). We did not use the AVA software cleaning filters that discard sequences under a fixed cut-off. Instead, the AVA alignments were extracted, truncated to the V3 *env* region, and gaps were removed. Reads with undetermined bases were discarded. Reads were then temporary translated into amino acid sequences in the right open reading frame in order to discard V3 sequences considered as non-functional if (i) they did no start and end with the cysteine required for the disulfide bound maintaining the V3 loop; (ii) they were not 32–38 amino-acids long; (iii) they contained a stop codon; (iv) they were not sufficiently identical with the V3 consensus at three typical motifs: a “CTRP”-like signature at the N-terminus (V3 residues 1–4), a “GPGR”-like signature at the hairpin crown (V3 residues 15–18), or a “QAHC”-like signature at the C-terminus (V3 residues 32–35). An identity of 0.5 and 0.75 was allowed for substitution and insertion/deletion of an amino-acid at any of the three signature motifs (Fig. 1). The biological filters mainly discard sequences with frame shifts due to insertions/deletions of nucleotides in homopolymeric regions or stop codons. This step removed 5.7% (mean) of V3 reads.

### Statistical filters to discard sequences with artifactual point mutations

The statistical filters assessed the probability that a sequence with a point mutation at a position of interest for predicting coreceptor usage would be artifactual or authentic. We first determined the frequency of artifactual V3 variants among the reads of 20 virus clones whose Sanger sequences were used as reference. The mean frequency of artifactual V3 variants was 0.646% [exact Poisson 99% confidence interval (CI), 0.00560–0.00742] of the reads. We defined the global error rate as the upper 99% confidence interval limit of this mean frequency of artifactual V3 variants. Based on this global error rate of 0.742% (μ), we estimated the expected number of artifactual sequences (*λ*) for *N* reads (*λ* = *N* * *μ*). We then used Poisson distribution to determine the minimum threshold above which a minor variant could be considered authentic for a given number of reads with *P* < 0.001. This fixed cut-off provided sensitivity thresholds from 1.7% for 1,000 reads to 1.14% for 5,000 reads.

**Figure 2:**
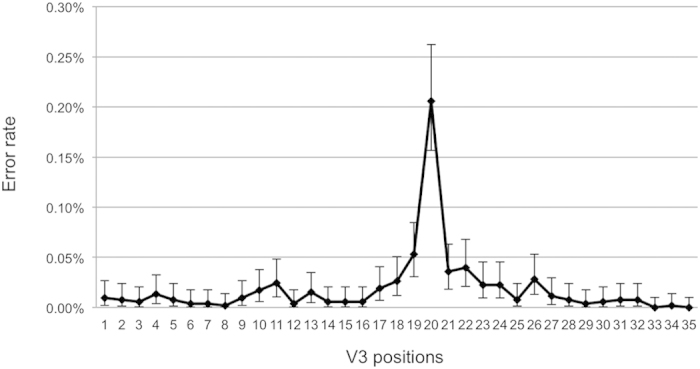
Error rate of amplification and ultra-deep pyrosequencing at each position of the V3 sequence. The mean error rate of amplification and pyrosequencing was estimated at each position of V3 by comparing the pyrosequencing reads to the Sanger sequences of 20 clones. The mean error rate is shown with exact Poisson 99% confidence interval at each position of the V3 sequence.

However, only a few key V3 amino-acid residues significantly influence the prediction of CXCR4 coreceptor usage by genotypic algorithms. As the errors generated during ultra-deep pyrosequencing are sequence-dependant, arbitrary fixed cut-offs are not ideal for distinguishing authentic variants from artifactual ones. We have determined position-specific error rates along the V3 sequence, defined as the upper 99% confidence interval limit (Poisson statistics) of the mean frequency of artifactual codons at each V3 position among the 20 virus clones. The error rate varied greatly along the V3 sequence (Fig. 2). V3 position 20 had the highest error rate, followed by positions 19, 22, 21, 26, 18, and 11. We determined the percentage at which a given V3 position contributed to the mean error rate along V3. We then attributed a weighted error rate (ratio to 0.02857 – value if errors occurred constantly along the 35 positions of V3 - multiplied by the global error rate of 0.00742) at each position. These weighted error rates were used to construct a sensitivity threshold matrix for each position of V3 to retain a minor virus variants harboring a point mutations as authentic for a given number of reads with *P* < 0.001 (Table 2).

### Genotypic prediction of HIV-1 tropism

The genotypic prediction of CXCR4-tropism for a given V3 sequence must take into account several amino-acids of interest. We identified the positions at which amino acid K, R, D and E were present within a given sequence. The detection thresholds at these particular positions were defined for a given number of reads. A single threshold was then defined for each putative CXCR4-using variant, based on the criteria of the combined 11/25 and net charge rule which are necessary and sufficient to predict CXCR4 tropism (Table 3). This was then used to retain a sequence predicted to be CXCR4-tropic whose frequency was above the specific threshold defined for this particular sequence at a given number of reads. The statistical filter for the sensitivity threshold of sequences predicted to be CCR5-tropic (*i.e.* those having no criteria of the combined 11/25 and net charge rule) was based on the global error rate of the whole V3 region (μ = 0.00742), as defined above.

### PyroVir software

**Figure 3:**
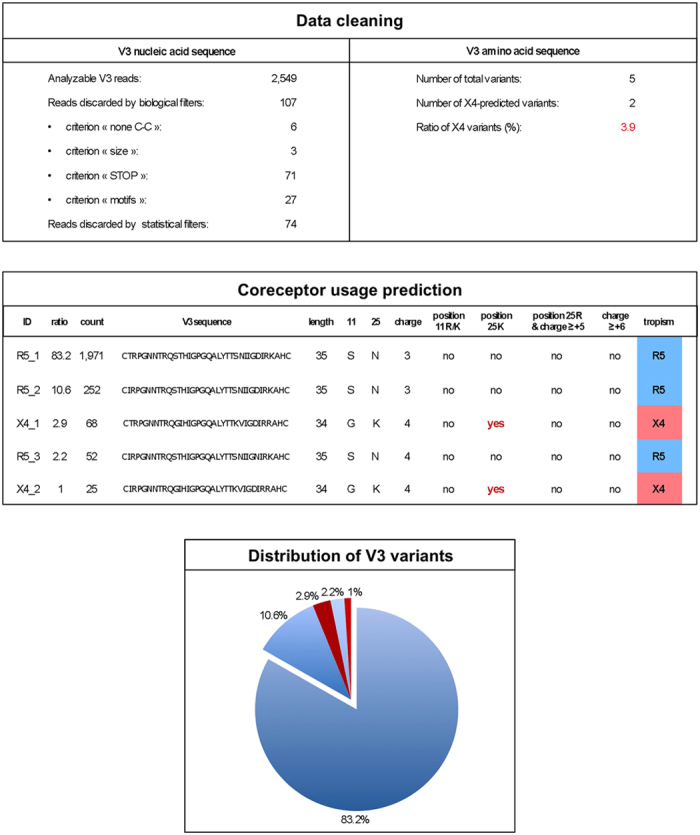
Example of PyroVir analysis for predicting HIV-1 quasispecies coreceptor usage. RNA was extracted from a plasma sample from an HIV-infected subject and submitted to nested RT-PCR amplification of V3 *env*, ultra-deep pyrosequencing, and PyroVir analysis. Ultra-deep pyrosequencing provided 2,549 analyzable V3 reads, of which 107 were discarded by the biological filter and and 74 by the statistical filter. 2 variants, one accounting for 2.9% and the other for 1% of the quasispecies, were predicted to use CXCR4.

This biological and statistical data cleaning strategy has been integrated in a program (PyroVir, IDDN FR.001.160011.000.S.P.2012.000.31230, Inserm-Transfert) that provides a fast, automated position-specific process for inferring HIV-1 tropism from V3 *env* 454 ultra-deep pyrosequencing data with improved detection of minor variants. The genotypic rule used to predict CXCR4-usage can be changed depending on the virus subtype, particularly for subtypes D and CRF01-AE for which we have developed specific algorithms^38^,^39^. An example of HIV-1 quasispecies coreceptor usage prediction by PyroVir is shown in Fig. 3. PyroVir is accessible at http://diag.ablsa.com/pyrovir/submit.php.

## Discussion

HIV-1 quasispecies coreceptor usage must be accurately determined before starting CCR5 antagonist-based antiretroviral therapy, as the presence of undetected minor CXCR4-using variants can lead to subsequent virological failure^24^,^25^. The development of gene therapy targeting CCR5 on hematopoietic stem cells in the quest for HIV cure would also require sensitive detection of CXCR4-using variants. Recombinant virus phenotypic entry assays are sensitive (0.3–0.5%) and considered to be the gold standard, but these assays are labour-intensive and expensive^8^,^14^. Genotypic methods based on bulk sequencing of V3 *env* combined with bioinformatics tools for inferring HIV-1 tropism are more rapid and more economical than phenotypic tests. But these simple genotypic assays are not sensitive enough to detect below 20% of minor CXCR4-using variants^21^,^22^,^23^. Ultra-deep pyrosequencing provides genotypic sensitivities similar to those of the current phenotypic assays, and also quantifies the proportion of each variant in the virus quasispecies. Ultra-deep pyrosequencing also has the advantage of using genotypic algorithms at a clonal level where genotype-phenotype correlations are better than for virus populations^21^. The PCR amplification step is an important potential source of artifacts, such as substitutions and recombinations, that can be minimized by an optimized amplification^40^,^41^,^42^. The *Taq* polymerase enzyme used has an important impact on the proportion of correct reads after sequencing^43^. But adequate representation of the virus quasispecies should also be preserved and this requires the use of a reduced number of PCR amplification cycles with a normalized virus input to ensure that the amplifications of both major and minor variants in the quasispecies are still in the logarithmic phase when the reaction is stopped.

Ultra-deep pyrosequencing of HIV-1 V3 *env* allows to detect low levels of CXCR4-using variants that current genotypic approaches miss. However, the extremely large data sets produced pose challenging computational problems, particularly the need to clean up the sequences by removing artifactual errors generated during amplification and pyrosequencing. Our PyroVir software rapidly and reliably predicts HIV-1 coreceptor usage from 454 ultra-deep pyrosequencing data. It has two modules. The first, biological filters, discard artifactual and non-functional sequences, particularly those due to frame-shifts generated by insertions or deletions of nucleotides in homopolymeric regions or stop codons. The second, statistical filters based on Poisson distribution, discard artifactual point mutations. This method is position-specific and does not use arbitrary fixed cut-offs to discard sequences with artifactual point mutations at V3 positions of interest as the errors generated during ultra-deep pyrosequencing are sequence-dependant. We found that the error rate of ultra-deep pyrosequencing varied along the V3 sequence, being maximum around V3 position 20. PyroVir automatically determines the sensitivity threshold for a given number of reads at each of the V3 positions involved in predicting CXCR4-usage, and then retains the highest threshold of the critical positions necessary and sufficient for predicting that a sequence is CXCR4-using. Subtype-specific algorithms could be used, especially for non-B subtypes such as subtypes D and CRF01-AE^38^,^39^,^44^. Arbitrary fixed cut-offs are currently used for cleaning up ultra-deep pyrosequencing data, usually 1 to 2%, below which sequences are discarded. These cut-offs have been determined based on rough error rates of ultra-deep pyrosequencing, and virological response rates in clinical studies with a limited number of patients^34^. Our results show that position-specific thresholds must be used to reliably detect minor variants. The sensitivity threshold varies greatly for a given number of reads (0.4 to 6.2% for 5,000 reads), depending on the V3 positions critical for CXCR4 usage. Therefore, using a fixed cut-off could result in a lack of sensitivity for some variants if the V3 positions involved have low error rates or even to false positives if the error rate is high.

The clinical relevance of minor variants is a matter of debate but should be distinguished from the analytical sensitivity of the method used. Previous studies reported cases of virological failure under CCR5 antagonists due to minor variants <1%^24^, while other found that a 2% cut-off optimally predicted the clinical response^34^. Most of the data on the relevance of minor variants in HIV drug resistance have been reported in studies of non-nucleoside reverse transcriptase inhibitors (NNRTIs). Minor variants at frequencies of <0.5% have been demonstrated to have a clinical impact on the virological response^45^. It has also been suggested that absolute numbers of resistant viruses are more clinically relevant than their frequencies for assessing the risk of subsequent virological failure. The measured frequency of viruses harboring a mutation associated with drug resistance should thus be multiplied by the plasma virus load to determine the absolute numbers of resistant viruses per mL of plasma. Minor resistant viruses in concentrations of 10–99 copies/mL were found to have a statistically significant impact on the virological response to NNRTIs, while concentrations of 1–9 copies/mL did not^45^. Interpretations of the impact of minor resistant viruses on the virological response are subject to additional caveats, notably the fitness and infectivity of the minor resistant viruses, and the effectiveness of the other molecules included in the combined antiretroviral regimen given to the subject. Analysis of the response to CCR5 antagonists is further complicated by the antiviral effect of CCR5 antagonists on R5 × 4 dualtropic variants in which CCR5 usage is more important than that of CXCR4 (<< dualR5 >> variants)^46^.

To summarize, we have developed an optimized process for the undistorted amplification and ultra-sensitive characterization of the coreceptor usage of HIV-1 quasispecies using ultra-deep pyrosequencing. This automated approach uses biological filters to discard artifactual or non-functional V3 sequences followed by statistical filters to determine position-specific sensitivity thresholds to identify authentic sequences with point mutations at V3 positions of interest.

## Methods

### Sample processing

The HIV-1 RNA in plasma samples was quantified with COBAS Ampliprep/COBAS TaqMan HIV-1 test version 2.0 (Roche). Plasma samples with a virus load of <10,000 copies/ml were ultracentrifuged at 20,000 g for 2h to concentrate the virus and RNA was extracted using the QIAamp Viral RNA Mini Kit (Qiagen). The initial input was adjusted to 2,000–3,000 copies of virus per PCR reaction, performed in duplicate, to avoid sampling bias. A lower input (300 copies) results in greater variability in the initial RT-PCR amplification for viruses at low frequencies and a risk of resampling (Supplementary Fig. S2).

### Amplification steps

A 1009-bp nucleotide fragment encompassing the V1-V3 *env* region of HIV-1 RNA was amplified by RT-PCR. The linearity of the PCR amplification process was checked by comparing the proportions of X4 (LAI, GenBank accession no. K02013.1) and R5 (JR-CSF, GenBank accession no. M38429.1) clones in the pyrosequencing output with the input of X4:R5 virus clones mixed in proportions of 0:100, 0.5:99.5, 1:99, 5:95, 20:80, 50:50, 75:25, and 100:0, adjusted to a total input of 2,000–3,000 copies of RNA, and submitted to multiple parallel PCR amplifications with various numbers of cycles. The resulting optimized process used the SuperScript III One-Step RT-PCR System (Invitrogen) for RT-PCR with the following conditions: 60 min at 55 °C; 2 min at 94 °C; 30 s at 94 °C, 30 s at 55 °C, and 1 min 30 s at 68 °C for 10 cycles; the annealing temperature was then increased to 58 °C for the next 24 cycles, without a final extension step. The following primers were used: forward 5′- CCACCACTCTATTTTGTGCATCA-3′; reverse 5′- CAGTAGAAAAATTCCCCTCCACA-3′. The nested PCR of V3 *env* was performed on pooled products of the first amplification with the Phusion High-Fidelity DNA Polymerase (Thermo Scientific) in the presence of DMSO (3%) as follows: 30 s at 98 °C; 10 s at 98 °C, 30 s at 55 °C, and 20 s at 72 °C for 10 cycles; the annealing temperature was then increased to 60 °C for the next 15 cycles without a final extension step. The number of PCR cycles was limited to 25 to ensure that the amplification remained linear, as described above. The nested primers were specific fusion primers needed to fuse to the emulsion PCR beads required by the 454 technology. They also included a 4-nucleotide sequencing key “TCAG” to identify the DNA library, 10-nucleotide multiplex identifiers (MIDs) used as a DNA barcode to identify samples after sequencing was complete, and a V3-spanning degenerate sequence (forward 5′- ACAATGYACACATGGAATTARGCCA -3′; reverse 5′- AGAAAAATTCYCCTCYACAATTAAA -3′). The amplified PCR products were analyzed using a LabChip GX (Caliper) and then purified using Agencourt Ampure PCR Purification beads (Beckman Coulter) to remove small (<300 bp) fragments. The purified PCR products were the quantified using a Quant-iT Picogreen dsDNA Assay Kit (Invitrogen) on a LightCycler 480 (Roche) and diluted to a concentration of 1 × 109 molecules/μl.

### V3 *env* ultra-deep pyrosequencing

Ultra-deep pyrosequencing was performed on a 454 GS Junior. PCR amplicons were combined and clonally amplified on DNA capture beads in water-in-oil emulsion micro-reactors at a ratio of 0.4 copies per capture bead. A total of 500,000 enriched-DNA beads were thus deposited in the wells of a full GS Junior Titanium PicoTiterPlate device and pyrosequenced in both forward and reverse directions. Bases were flowed sequentially and always in the same order (TCAG) across the wells of the PicoTiterPlate device during a 10-hour sequencing run generating long (500 bp) sequences.

### Genotypic prediction of HIV-1 coreceptor usage from V3 ultra-deep pyrosequencing data

The sequences of the V3 *env* regions were first processed using GS Amplicon Variant Analyzer (AVA) software, version 2.5 p1 (Roche). This software extracts sequences from the standard flowgram format (SFF) files generated after pyrosequencing and automatically assigns each read to the proper sample by looking for the MIDs located at both ends of V3. Only sequences with an average phred equivalent quality score >Q30 were conserved. Moreover, only sequences that had been read in both senses were used for further analyses. The MIDs and primer sequences within the read have also to be complete without mismatch. Moreover, the reads have to match the full-length amplicon The sequence reads were aligned with the BaL consensus sequence (GenBank accession no. AY426110.1) and processed using an in-house automated data cleaning strategy (see Results) rather than the AVA filters. We used the combined 11/25 and net charge rule to infer the tropism of each virus clone from the V3 amino acid sequence. It requires one of the following criteria for predicting the CXCR4 coreceptor usage of HIV-1 subtype B^21^,^23^: (i) an R or K at position 11 of V3 and/or a K at position 25; (ii) an R at position 25 of V3 and a net charge of at least +5; and (iii) a net charge of at least +6. The V3 net charge was calculated by subtracting the number of negatively charged amino acids (D and E) from the number of positively charged ones (K and R). Subtype-specific algorithms derived from the combined 11/25 and net charge rule have been developed for subtypes D, and CRF01-AE^38^,^39^.

### Determining the global error rate of ultra-deep pyrosequencing of V3

We estimated the frequency of errors introduced during V3 amplification and GS Junior pyrosequencing by comparing the pyrosequencing reads to the Sanger sequences of 20 plasmid clones of *env* obtained from HIV-1 subtype B primary isolates. We first determined the frequency of artifactual V3 variants among the reads of each virus clone. The global error rate of ultra-deep pyrosequencing was then defined as the upper limit of the 99% confidence interval (Poisson statistics) of the mean frequency of artifactual V3 variants among the reads of the 20 clones.

Poisson distribution was applied to this global error rate to assess the risk of an artifactual V3 sequence being an authentic variant. We calculated the probability that a minor variant with *n* occurrences in *N* reads would occur *n* or more times if it was an error, using the following formula:


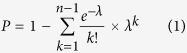


Here, *λ* is the expected number of artifactual sequences given *N* reads and is calculated by *λ* = *N* * *μ*, with *μ* being the global error rate (defined above). Only those variants whose frequency of occurrence yielded a *P* value of <0.001 according to the Poisson model were considered authentic.

### Determining the position-specific error rates of ultra-deep pyrosequencing along the V3 sequence

As the errors generated during ultra-deep pyrosequencing are sequence-dependant, we determined specific error rates at each position in V3. We measured the mean codon error rate among the 20 clones at each V3 position. The position-specific error rates were then defined as the upper limit of the 99% confidence interval (Poisson statistics) of the mean frequency of artifactual codons among the 20 clones at each position of V3. We then determined weighted error rates to construct a sensitivity threshold matrix at each position of V3 to identify authentic virus variants harboring a point mutations for a given number of reads with *P* < 0.001.

### Sensitivities of phenotyping and ultra-deep pyrosequencing for detecting and quantifying minor CXCR4-using variants

We assessed the capacity of ultra-deep pyrosequencing to detect and correctly quantify minor CXCR4-using variants in a virus population of CCR5-using variants using artificial mixtures of X4 and R5 virus clones that were phenotyped in parallel using the ultrasensitive TTT phenotypic assay^8^. Three artificial mixtures of X4 and R5 virus clones were used: LAI (GenBank accession no. K02013.1, X4 phenotype) and JR-CSF (GenBank accession no., R5 phenotype); AFG04 (GenBank accession no. DQ136796.1, X4 phenotype) and AFG01 (GenBank accession no. DQ136807.1, R5 phenotype); CHS02 (GenBank accession no. DQ136867.1, X4 phenotype) and CHS11 (GenBank accession no. DQ136859.1, R5 phenotype). AFG and CHS are primary HIV-1 isolates that had previously been cloned and phenotyped for CCR5 anf CXCR4 coreceptor usage^47^. The HIV-1 RNA in culture supernatants of the pure R5 and X4 clones was quantified using the COBAS Ampliprep/COBAS TaqMan HIV-1 test version 2.0 (Roche), and then mixed in defined proportions of X4:R5 viruses (0:100; 0.5:99.5; 1:99; 5:95; 20:80; 50:50; 75:25; and 100:0, each with 2–3 replicates). RNA was then extracted, adjusted to a total of 3,000 virus copies/reaction in triplicate, and submitted to ultra-deep pyrosequencing and phenotyping in parallel.

### Statistics

Poisson statistics were calculated using R version 3.0.0.

### Langage programming

PyroVir was written in the Java programming language and run with the Java 6.25 software.

## Additional Information

**How to cite this article**: Jeanne, N. *et al.* Position-specific automated processing of V3 *env* ultra-deep pyrosequencing data for predicting HIV-1 tropism. *Sci. Rep.* **5**, 16944; doi: 10.1038/srep16944 (2015).

## Supplementary Material

Supplementary Information

## Figures and Tables

**Table 1:** Quantifying X4 variants in HIV-1 quasispecies by ultra-deep pyrosequencing.

X4 input (%)	X4 quantification in X4:R5 virus mixtures (%)								
	LAI:JR-CSF*			AFG4:AFG1*			CHS2:CHS11*		
0.5	0.5	0.0	0.6	0.7	0.7	0.0	0.0	0.0	0.0
1.0	1.7	0.0	1.0	0.9	1.1	1.0	0.6	0.5	0.0
5.0	2.3	2.6	2.0	5.3	5.4	7.1	5.0	4.2	5.3
20.0	9.2	1.8	2.5	21.4	23.4	—	30.0	25.2	—
50.0	57.8	43.9	40.7	53.2	56.2	—	56.2	53.1	—
75.0	73.6	78.3	79.3	74.3	70.8	—	80.6	81.6	—
100.0	100.0	100.0	100.0	100.0	100.0	—	100.0	100.0	—

^*^Each virus mixture was assessed in triplicate or duplicate beyond 5%

**Table 2:**
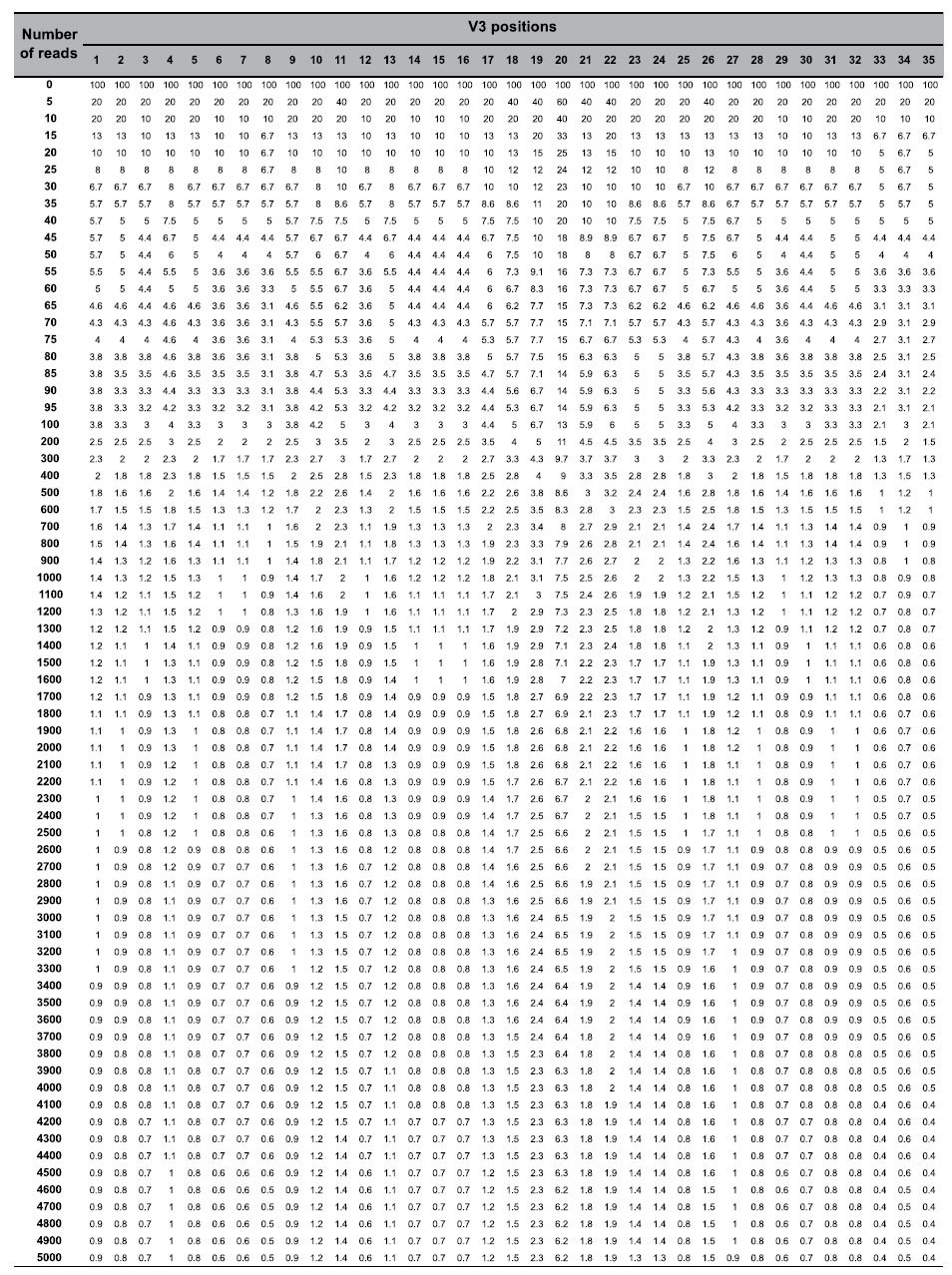
V3 position-specific matrix of sensitivity thresholds.

**Table 3:** Determining a single sensitivity threshold necessary and sufficient for predicting CXCR4-usage according to the combined 11/25 and net charge rule.

case	Criteria of the combined 11/25 and net charge rule for HIV-1 subtype B						retained threshold
			25R and net charge* ≥ +5		net charge* ≥ +6		
	11R or 11K	25K	25R and net charge = +5	25R and net charge > +5†	net charge = +6	net charge > +6‡	
1	x						position 11
2		x					position 25
3			x				maximum§
4				x			maximum†,§
5					x		maximum¶
6						x	maximum‡,¶
7	x	x					minimum#
8	x		x				minimum§,||
9	x			x			minimum†,§,||
10	x				x		minimum¶,**
11	x					x	minimum‡,¶,**
12		x			x		minimum¶,††
13		x				x	minimum‡,¶,††
14	x	x			x		minimum¶,‡‡
15	x	x				x	minimum‡,¶,‡‡

^*^The V3 net charge was calculated by subtracting the number of negatively charged amino acids (D and E) from the number of positively charged ones (K and R).

^†^The most pejorative positions harboring a positively charged residue were eliminated to reach the criterion << 25R and net charge = +5 >>. Alternatively, position 25 can be eliminated to arrive at the criterion << net charge = +6 >> if more favorable.

^‡^The most pejorative positions harboring a positively charged residue were eliminated to arrive at the criterion << net charge = +6 >>.

^§^The most pejorative threshold between that of position 25 and those required for a net charge of +5 is used.

^¶^The most pejorative threshold of the positions harboring a positively charged residue was used.

^#^The least pejorative threshold between those of positions 11 and 25 is used.

^||^The least pejorative threshold between that of position 11 and those required for the << 25R and net charge = +5 >> criterion is used.

^**^The least pejorative threshold between that of position 11 and those required for a net charge of +6 is used.

^††^The least pejorative threshold between that of position 25 and those required for a net charge of +6 is used.

^‡‡^The least pejorative threshold between those of positions 11, and 25, and those required for a net charge of +6 is used.
