# Supplementary material for: Position-specific automated processing of V3 env ultra-deep pyrosequencing data for predicting HIV-1 tropism
Source: Sci Rep. 2015 Nov 20;5:16944. doi: 10.1038/srep16944 (PMC4653658; doi:10.1038/srep16944)
Supplement: Supplementary Information [file srep16944-s1.pdf]

Position-specific automated processing of V3 *env* ultra-deep pyrosequencing data for predicting HIV-1 tropism

Nicolas Jeanne, Adrien Saliou, Romain Carcenac, Caroline Lefebvre, Martine Dubois, Michelle Cazabat, Florence Nicot, Claire Loiseau, Stéphanie Raymond, Jacques Izopet, & Pierre Delobel

Supplementary Fig. S1

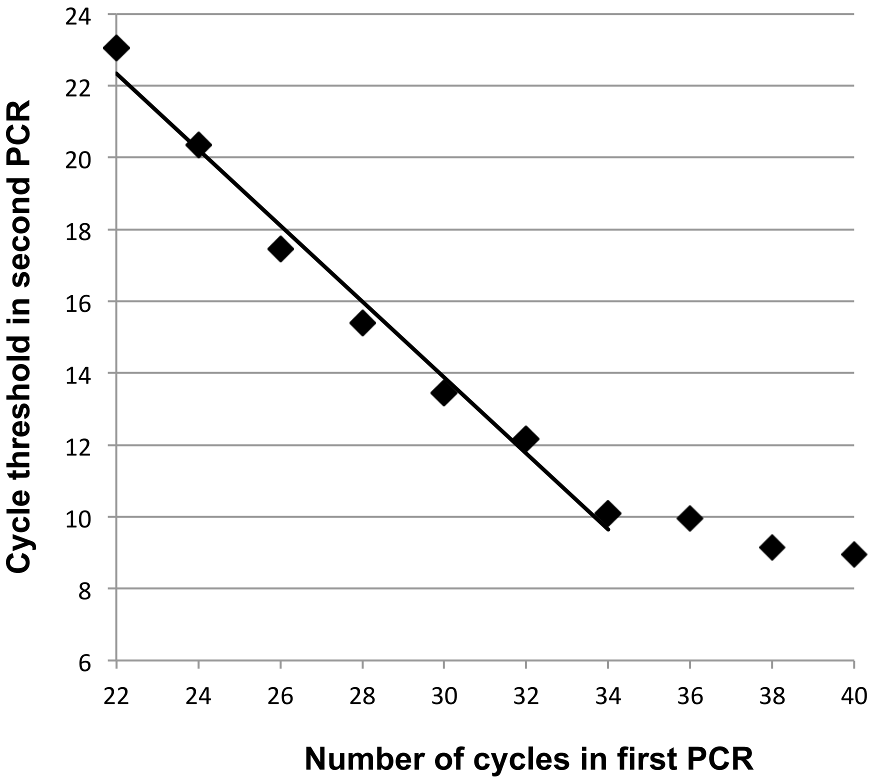

Fig. S1. Linearity of PCR amplification
The first-round PCR was stopped every 2 cycles from 22 to 40 cycles and the amplified HIV-1 DNA content was quantified by real-time PCR. The HIV-1 DNA contents of the replicates were then quantified by real-time PCR. The detection threshold in the second reaction was related to the number of amplification cycles in the first reaction to determine the cycle number at which the first amplification remained linear. Our results show that the PCR amplification remains linear until 34 cycles. These results were reproduced in four independent experiments.

Supplementary Fig. S2

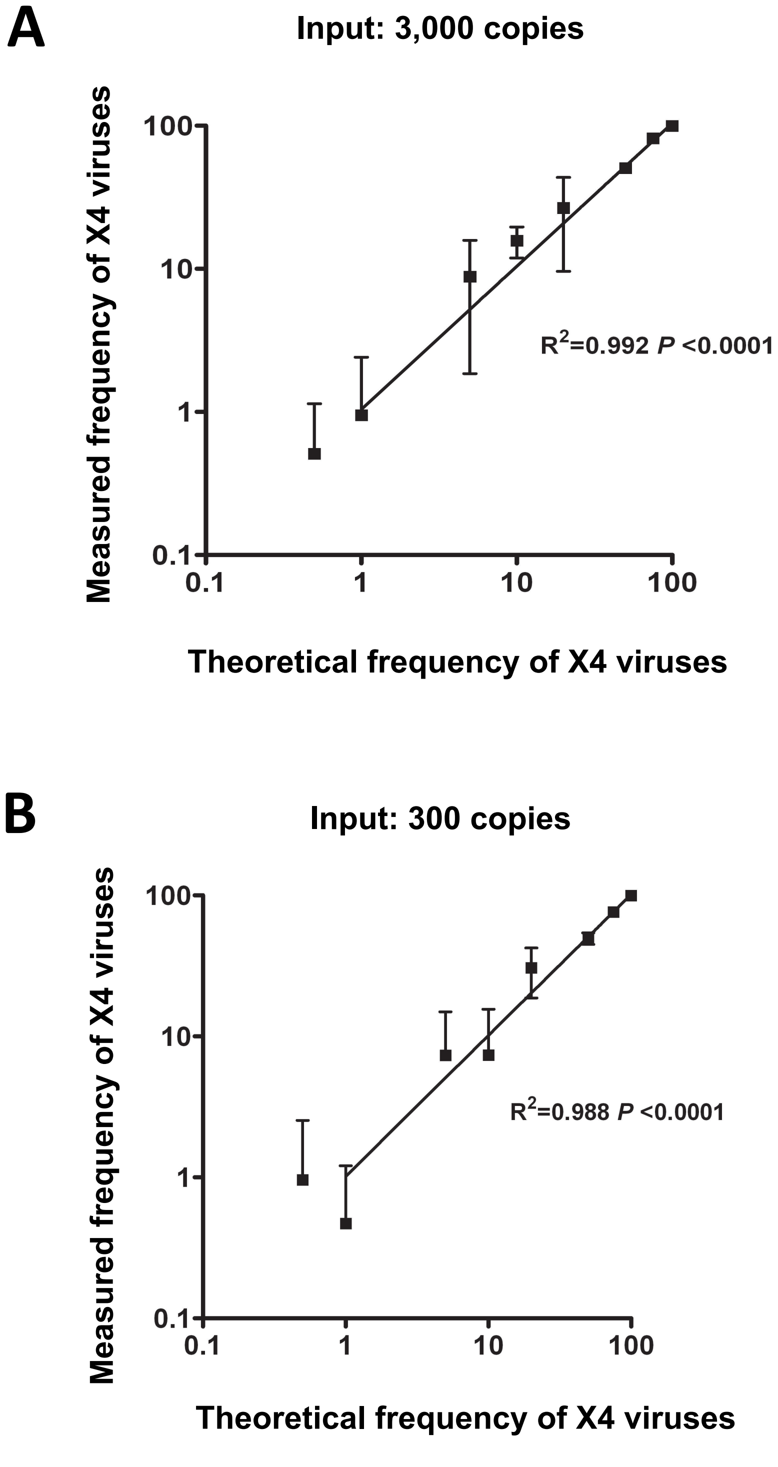

Fig. S2. Impact on virus input on measured frequencies of X viruses
X4 (LAI) and R5 (JR-CSF) virus clones were mixed in proportions of 0:100, 0.5:99.5, 1:99, 5:95, 10:90, 20:80, 50:50, 75:25 and 100:0 and adjusted to a total input of 3,000 or 300 RNA copies. The measured frequencies of X4 viruses by ultra-deep pyrosequencing was compared to the theoretical frequencies of X4 viruses for a total input of 3,000 and 300 RNA copies.
